# Distinct Redox Profiles of Selected Human Prostate Carcinoma Cell Lines: Implications for Rational Design of Redox Therapy

**DOI:** 10.3390/cancers3033557

**Published:** 2011-09-13

**Authors:** Luksana Chaiswing, Weixiong Zhong, Terry D. Oberley

**Affiliations:** 1 Department of Pathology and Laboratory Medicine, School of Medicine and Public Health, University of Wisconsin, 1111 Highland Ave., WIMR 7168, Madison, WI 53705, USA; E-Mails: lchaiswing@wisc.edu (L.C.); wzhong3@wisc.edu (W.Z.); 2 Pathology and Laboratory Medicine Service, William S. Middleton Memorial Veterans Hospital, Rm A-35, 2500 Overlook Terrace, Madison, WI 53705, USA

**Keywords:** ROS/RNS, redox state, cell growth, cell viability, cell invasion, PrEC, PC3, RWPE1, WPE1-NA22, WPE1-NB26, LNCaP, LNCaP-C4-2 cell lines

## Abstract

The effects of several cancer chemotherapeutic drugs and radiation are mediated, at least in part, by oxidative stress. To better understand this process, we analyzed certain biochemical properties affecting reduction-oxidation (redox) balance in normal prostate epithelial cells and several prostate cancer cell lines. Highly aggressive androgen-independent prostate cancer PC3 cells demonstrated significantly higher levels of total antioxidant capacity (AC) and intra- and extracellular glutathione (GSH)/glutathione disulfide (GSSG) ratios when compared with normal prostate epithelial PrEC cells. WPE1-NB26 cells, a prostate cancer cell line derived from immortalized RWPE1 human prostate epithelial cells, demonstrated significantly higher levels of total AC and intra- and extracellular GSH/GSSG ratios, but lower levels of intracellular reactive oxygen/nitrogen species and lipid peroxidation compared with RWPE1 cells. LNCaP-C4-2 cells, a more aggressive prostate cancer derived from less aggressive androgen-responsive LNCaP cells, exhibited higher levels of AC and extracellular GSH/GSSG ratio when compared to LNCaP cells. Specific cell types showed distinct cytotoxic responses to redox-modulating compounds. WPE1-NB26 cells were more sensitive to phenethyl isothiocyanate and tumor necrosis factor (TNF) than RWPE1 cells, while PC3 cells were more sensitive to TNF than PrEC cells. These results are consistent with the hypothesis that cancer cell redox state may modulate responses to redox-modulating therapeutic regimens.

## 1. Introduction

The term cellular redox state refers to the balance between oxidizing and reducing equivalents in the cell. Primary determinants of cell redox state are levels of reactive oxygen species/reactive nitrogen species (ROS/RNS) relative to the levels of antioxidant enzymes (AEs) and small molecular weight antioxidants. ROS/RNS include both oxygen/nitrogen radicals and nonradicals that are oxidizing agents and/or are easily converted into radicals [1]. An example of ROS is superoxide radical (O_2_^•−^), which is known to either oxidize biomolecules, such as lipids and proteins, or result in the production of more reactive chemical species such as hydrogen peroxide (H_2_O_2_), hydroxyl radical, and peroxynitrite. O_2_^•−^ is produced during mitochondrial respiration and by a family of membrane-bound enzymes known as nicotinamide adenine dinucleotide phosphate (NADPH) oxidases (NOX) [2]. The RNS include nitric oxide (^•^NO) and species resulting from ^•^NO's rapid reactions with other molecules. The sources of ^•^NO production are the nitric oxide synthase (NOS) isoforms, including neuronal NOS (nNOS), endothelial NOS (eNOS), and inducible NOS (iNOS) [3].

The intracellular antioxidant proteins in mammalian cells are usually classified as primary or secondary. Primary AEs include superoxide dismutases [SODs; manganese SOD (MnSOD), copper, zinc SOD (CuZnSOD), and extracellular SOD (EC-SOD)], catalase (CAT), and glutathione peroxidases (GPxs). The SODs convert O_2_^•−^ to H_2_O_2_ and can have both antioxidant (reduction of O_2_^•−^) and prooxidant effects (production of H_2_O_2_), while CAT and GPxs convert H_2_O_2_ to water. Additionally, GSTs are directly responsible for the elimination of electrophilic oxidants at the expense of glutathione (GSH) [4]. The secondary antioxidant proteins do not act directly on RNS/ROS but are involved in generation of intracellular reducing agents or electron donors, such as the reduced form of GSH, cysteine, and NAD(P)H. The secondary antioxidant proteins include glutathione reductase (GR), glucose-6-phosphate dehydrogenase (G6PD), glutathione S-transferases (GSTs), gamma-glutamyl transpeptidase (GGT), gamma glutamylcysteine synthase (GCS), thioredoxin reductases (TRs), thioredoxins (Trxs), and peroxiredoxins (Prxs). Some antioxidant proteins are highly compartmentalized, including MnSOD and TR2, which are located in mitochondria, and Trx1, which is located in the nucleus and cytoplasm [5]. Other antioxidant proteins are located in several subcellular compartments, examples being GPxs, Prxs, and CuZnSOD [6].

Extracellular/microenvironmental redox states are demonstrated to be involved in several physiological and pathological events. Extracellular redox state is determined at least in part by the following factors [7]: (1) redox modulating proteins located in the plasma membrane such as NADPH oxidase; (2) redox modulating proteins located outside cells such as EC-SOD; (3) thiol/disulfide couples such as cysteine (Cys)/cystine (CySS); (4) ROS/ RNS that are capable of traveling across cell membranes, such as H_2_O_2_; (5) extracellular free radical damage products, including protein carbonyls, and (6) extracellular repair systems, such as protein disulfide isomerase. These molecules are the machinery that maintains redox homeostasis in the extracellular space/microenvironment.

Many studies have demonstrated that both intra- and extracellular redox states play an important role in biological processes of both normal and cancer cells, examples being cell proliferation/cell cycle progression [8,9], transcription/translation [10], signal transduction [11], and apoptosis [12]. At low levels, ROS are involved in the regulation of gene expression by direct participation in cell signal transduction. In addition, many transcription factors have been identified to be redox-sensitive, examples being nuclear factor kappaβ (NF-*k*β) [13], activator protein 1 [14], and p53 [15].

Prostate cancer is the most commonly diagnosed non-skin cancer in men in the United States. Accumulating evidence has implicated the involvement of oxidative stress in the development and progression of prostate cancer [16,17]. Our laboratory used antibodies specific to oxidative damage products to analyze differences in redox state in subcellular organelles in comparisons of normal human prostate, primary prostate cancer, and metastatic prostate cancer tissues, and found that levels of mitochondrial MnSOD protein and nuclear levels of the oxidative damage products 8-hydroxy-2′-deoxyguanosine (8OHdG), 4-hydroxy-2-nonenal protein adducts, and 3-nitrotyrosine were greater in metastasic cancers compared to primary human prostate cancers [18]. In addition, we demonstrated differences in intracellular redox state between a more aggressive human metastatic prostate cancer cell line (PC3) and a less aggressive prostate cancer cell line (LNCaP) during cell proliferation [19]. PC3 demonstrated lower levels of intracellular ROS/RNS and cytoplasmic lipid peroxidation (LPO) and greater levels of nuclear 8OHdG and intracellular GSH/glutathione disulfide (GSSG) ratio than LNCaP cells. In addition, EC-SOD, transferrin (TF), TR1, GCS, G6PD, and GSTP1 protein levels were higher in PC3 cells than in LNCaP cells. Thus, PC3 cells have greater antioxidant capacity (AC) than LNCaP cells, allowing greater cell survival after treatment with redox modulating compounds such as diphenyleneiodonium (DPI, an inhibitor of NADPH oxidase and other flavin-containing molecules) or H_2_O_2_. We also demonstrated that LNCaP cells required a prooxidant state for cell proliferation, while PC3 cells proliferated with low levels/in the absence of such a prooxidant state. The results suggest the possibility of intracellular redox state modulation being used as a therapeutic tool to inhibit prostate cancer cell proliferation. Recent studies in our laboratory demonstrated that modulation of extracellular redox state of a prostate cancer cell line by overexpression of EC-SOD resulted in inhibition of cell invasion and decreased matrix metalloproteinase (MMP) and membrane type 1 MMP (MT1-MMP) enzymatic activities. Inhibition of cell invasion was demonstrated to occur in aggressive prostate cancer WPE1-NB26 cells, but not immortalized RWPE1 human prostate epithelial cells [20]. These results suggest that there are shifts in intra- and extracellular redox states as cancer progresses to a more aggressive state. These shifts are postulated to promote abnormal behavior of cancer cells.

Our overall hypothesis is that redox state in specific subcellular compartments is intimately linked to regulation of cancer cell behavior, including growth, invasiveness, and metabolism. The present study specifically focused on intra- and extracellular redox states in various prostate cancer cell lines. Three model systems were used: normal prostate epithelial PrEC cells compared with highly aggressive androgen-independent PC3 prostate cancer cells; immortalized non-malignant human prostate epithelial RWPE1 cells compared with its derived isogenic highly aggressive malignant variants (WPE1-NA22 and WPE1-NB26); and less aggressive androgen-responsive LNCaP prostate cancer cells compared with its derived more aggressive LNCaP-C4-2 cell line. Our results consistently demonstrated that each prostate cancer cell line exhibited different redox characteristics. The significant differences in intra- and extracellular redox states correlated with different responses of each cell type to anti-cancer drugs or compounds. Our findings provide new insights into the possible usefulness or dangers of prooxidant or antioxidant agents as cancer therapeutic drugs; specifically, therapeutic results with these compounds will depend on the specific intra- and extracellular redox biochemistries of the cancer cell type that is the target of therapy.

## 2. Results and Discussion

The present study focused on intra- and extracellular redox states in normal/immortalized prostate epithelial cells and specific prostate cancer cell lines.

### 2.1. PrEC and PC3 Cells

#### 2.1.1. Redox Characteristic of PrEC *versus* PC3 Cells

**Figure 1.**
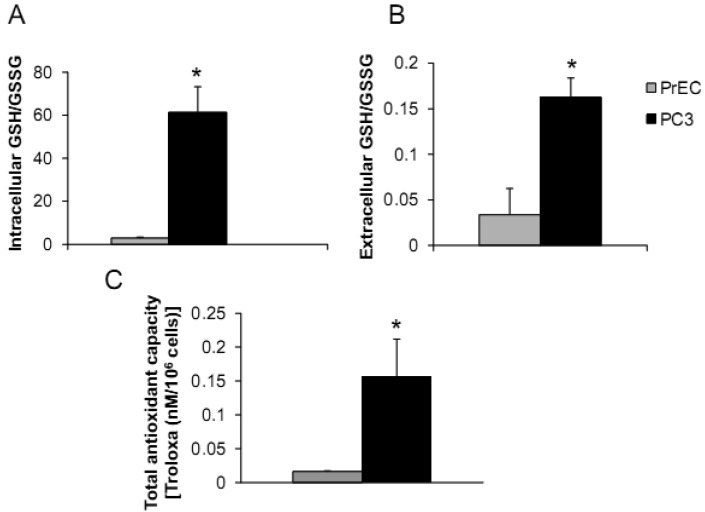
Analysis of redox state in PrEC *vs.* PC3 cells. PrEC or PC3 cells were cultured in prostate epithelial growth medium (PrEGM) or RPMI 1640 media for 24 h. Cells and conditioned media were collected for analysis. (**A**) Intracellular GSH/GSSG ratio; (**B**) Extracellular GSH/GSSG ratio; (**C**) Total antioxidant capacity. * P-value ≤ 0.05.

The androgen-independent PC3 cell line has high metastatic potential and was derived from a poorly differentiated lumbar vertebral metastasis of a 62-year-old Caucasian [23,24]. PC3 cells were able to colonize human bone implants after intravenous injections of tumor cells into severe combined immunodeficient mice [25]. The aggressiveness and redox profiles of PC3 cells were previously documented in our laboratory and demonstrated that PC3 cells have low levels of ROS/RNS and high intracellular GSH/GSSG ratio [19]. Herein, we compared PC3 cells with normal prostate epithelial PrEC cells, as shown in Figures 1A–C.

PC3 cells demonstrated significantly greater levels of intra- and extracellular GSH/GSSG ratios and total intracellular AC when compared to PrEC cells. Powolny and Singh assessed gene expression by real-time PCR using human oxidative stress and antioxidant defense RT^2^ profiler and demonstrated up-regulation of several gene expression levels in PC3 cells in comparison to PrEC cells including GPx4, Prx1, Prx2, Prx6, CuZnSOD, MnSOD, TR1, and TR2 [26]. Additionally, levels of glutathione-related gene expression were down-regulated in PC3 cells including GPx6 and 7 [26]. Differences of intra- and extracellular redox states of prostate cancer cells may correlate with cancer phenotypics properties, including dysregulated cell growth and increased cell invasion.

#### 2.1.2. Effects of Tumor Necrosis Factor (TNF), TNF-Related Apoptosis Inducing Ligand (TRAIL), or Overexpression of MnSOD on PrEC or PC3 Cells

**Figure 2.**
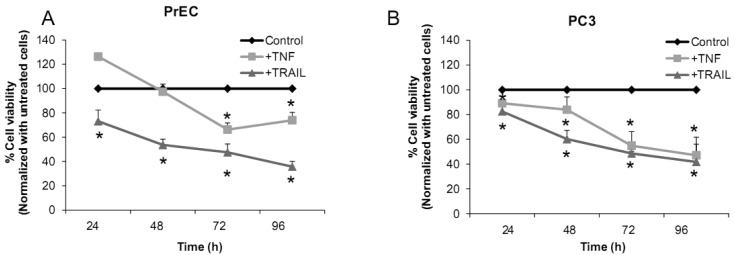

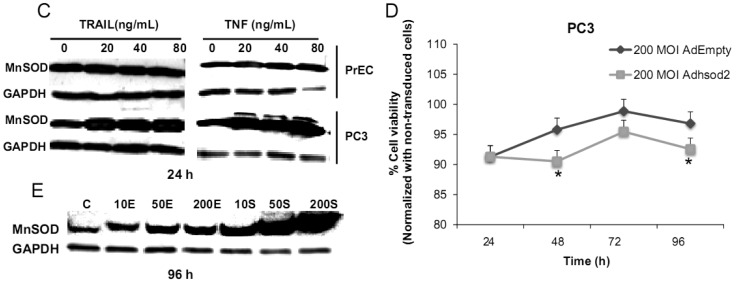
Effects of TNF, TRAIL, or overexpression of MnSOD on cell viability and immunoreactive MnSOD protein levels. (**A**) PrEC or (**B**) PC3 cells were treated with 40 ng/mL TNF or TRAIL for 24, 48, 72, or 96 h. Cell viability using 3-[4,5-dimethylthiazol-2-yl]-2,5-diphenyl tetrazolium bromide (MTT) assays were performed; (**C**) MnSOD protein expression in PrEC or PC3 cells after treatment with 0, 20, 40, or 80 ng/mL TNF or TRAIL for 24 h; (**D**) PC3 cells were transduced with 200 multiplicity of infectivity (MOI) adenoviral vectors containing human MnSOD cDNA (Adh*sod2*) or without cDNA insert (AdEmpty) for 24 h, MTT assays were performed every 24 h; (**E**) MnSOD protein expression in PC3 cells after transduction with Adh*sod2* or AdEmpty for 96 h. E = AdEmpty, S = Adh*sod2*, C = PC3 without adenoviral transduction. * P-value ≤ 0.05.

We have challenged PrEC or PC3 cells with TNF or TRAIL to study whether the differences in fundamental redox state of these two cell lines affect cell viability in response to these biologic modifiers. Treatment of PrEC or PC3 cells with 40 ng/mL TNF or TRAIL resulted in alterations in cell viability in comparison to untreated cells (Figures 2A,B). PC3 cells demonstrated more resistance to TRAIL-induced apoptosis than PrEC cells in the first 24 h of incubation. In contrast, TNF induced cell death in PC3 cells more than in PrEC cells at 24 h and 48 h. Induction of MnSOD protein expression levels were observed in PC3 cells treated with TNF or TRAIL at 24 h (Figure 2C). Jones *et al.* demonstrated that TNF and interlukin-1β induced MnSOD protein expression through NF-*k*β in mice [27].

We postulate that induction of MnSOD may induce H_2_O_2_ mediated cell death. H_2_O_2_ could possibly inactivate GSH/GSSG systems, resulting in accumulation of ROS/RNS. PC3 cells did not require high levels of ROS/RNS for cell proliferation [19]; thus, accumulation of ROS/RNS may induce oxidative stress with subsequent killing of cancer cells.

To gain mechanistic insights into the possible role of MnSOD in affecting viability of PC3 cells, a proof-of-principle analysis using an adenoviral expression vector containing human *sod2* showed that cell viability of PC3 cells was reduced by overexpression of MnSOD (Figure 2D); other studies demonstrated that overexpression of MnSOD in PC3 cells resulted in inhibition of PC3 cell proliferation by retarding G_1_ to S transition of the cell cycle [28]. Our laboratory has provided data in SV-40 transformed fibroblast cells that increased H_2_O_2_ within mitochondria resulted in inhibition of cell cycle progression [22]. The increased H_2_O_2_ levels can have positive or negative effects on cell cycle progression, depending on levels and the subcellular location of increased H_2_O_2_. We postulate that increased H_2_O_2_ in mitochondria inhibits cell cycle progression as a protective mechanism to prevent cell replication. In contrast, elevation of H_2_O_2_ at the cell surface largely mediated by growth factor mechanism(s) results in activation of cell cycle progression [29-31]. Alternatively, TNF may induce ROS mediated cell death via the activation of NOX1 [32]. It should be emphasized that gene therapy with MnSOD cDNA is not the only way to modulate MnSOD activity; herein, we demonstrated that biologic modifiers such as TNF or TRAIL induce endogenous MnSOD protein expression and thus offer a possible pathway for therapeutic intervention in cancer involving AE imbalance and subsequent increase in ROS. Notably, both PC3 and PrEC cell viabilities were significantly decreased following TRAIL treatment. It is possible that TRAIL induced apoptosis in these cells is not regulated by intra- or extracellular redox states and TRAIL may not be suitable for prostate cancer therapeutics since it also induced PrEC cell death [33].

### 2.2. RWPE1 Family

One of the problems in comparing PrEC and PC3 cells is that they are not syngeneic and were cultured in different media. Thus, intra- and extracellular redox differences may be present due to non-isogenic backgrounds and different media. To avoid this difficulty, we analyzed intra- and extracellular redox states in syngeneic prostate cancer cells WPE1-NA22 and WPE1-NB26 derived from RWPE1 cells. RWPE1 cells were produced by immortalization of epithelial cells isolated from the peripheral zone of a non-neoplastic adult human prostate with human papilloma virus-18 [34]. Tumorigenic cell lines were derived by exposure of RWPE1 to *N*-methyl-*N*-nitrosourea, selected and cloned *in vivo* and *in vitro*, and characterized by invasiveness, tumorigenicity, and pathology of the derived tumors [35]. The WPE1-NA22 cells (least malignant) form small, well-differentiated tumors, while WPE1-NB26 cells (most malignant) form large, poorly differentiated invasive tumors. These cell lines represent different stages of prostate cancer cell progression and are derived from the same cell type.

#### 2.2.1. Aggressive Characteristic of RPWE1, WPE1-NA22, or WPE1-NB26 Cells

**Figure 3.**
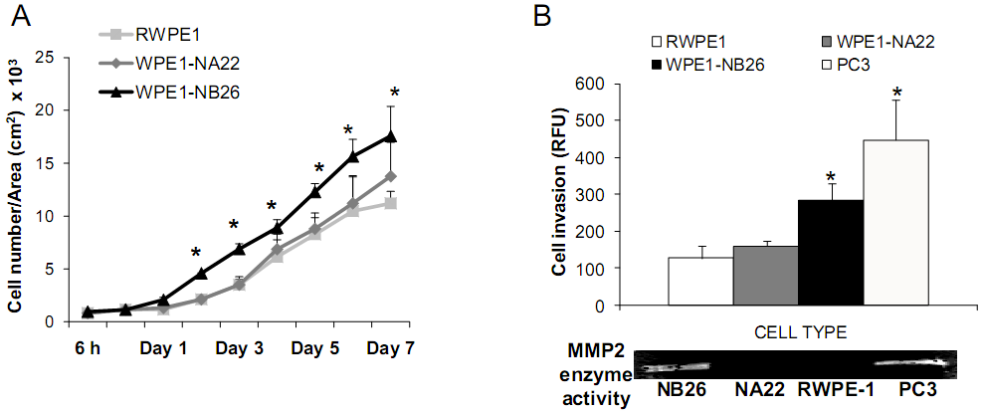

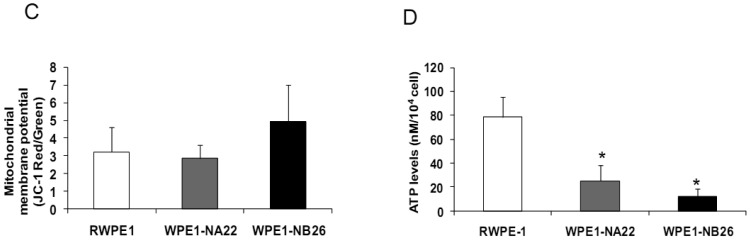
Biology of RWPE1, WPE1-NA22, or WPE1-NB26 cells. (**A**) Growth curves. Cells were seeded at 7 × 10^4^ cells/60 mm dish and counted every 24 h; (**B**) Invasion ability and MMP activity. Cells were cultured for 24 h in keratinocyte serum free medium (KSFM) without bovine pituitary extract (BPE) and recombinant epidermal growth factor (rEGF) prior to analysis as described in the Experimental section. Cells were collected for invasion assay and conditioned media were collected for MMP2 zymography. Gelatinolytic activities of MMP2 were detected as clear bands against a blue background. NB26 = WPE1-NB26 cells, NA22 = WPE1-NA22 cells. PC3 cells were used as a positive control; (**C**) Mitochondrial membrane potential. Cells (5 × 10^5^/mL) during log growth were collected, incubated with 10 μM JC-1 fluorescence dye, and analyzed by FACSCalibur flow cytometry with FL1 (green) and FL2 (red) channels. An increase in the ratio of JC-1 red to JC-1 green indicates an increase in mitochondrial membrane potential; (**D**) ATP levels. Cells during log phase were collected and homogenized. Cell homogenates were incubated with luciferase. ATP concentration was calculated using an ATP standard curve. RFU = Relative fluorescence units. * P-value ≤ 0.05 when compared to RWPE1 cells.

Aggressive/malignant characteristic of these cell lines are demonstrated in Figure 3. Cell growth analysis demonstrated that RWPE1 had the slowest growth [doubling time (Td) 31.9 ± 5.2 h], WPE1-NA22 was intermediate (Td 30.9 ± 4.7 h), whereas WPE1-NB26 had the fastest growth (Td 28.8 ± 4.6 h) (Figure 3A).

Aggressive cancer cell behavior is determined not only by the rate of cell growth, but also by the *in vitro* invasive ability of each cancer cell type. Invasion assays were performed to establish that analysis of RWPE1 family cell lines is a suitable model for characterizing cancer cell lines with different levels of aggressiveness. An *in vitro* invasion assay demonstrated that WPE1-NB26 had more invasive ability than the other two cell lines (Figure 3B).

Since invasive ability is correlated with levels of MMP, enzymes whose function primarily relates to degradation of extracellular matrix proteins necessary for cell invasion [36], we performed activity analyses of MMP2 and found that WPE1-NB26 had more MMP2 activity than the other two cell lines. PC3 cells were used as a positive control since it is the most aggressive cancer phenotype used in our current study (Figure 3B). We studied cell metabolic biochemistry and found that RWPE1 cells produced higher levels of ATP, whereas levels of mitochondrial membrane potential using JC-1 (5,5′,6,6′-tetrachloro-1,1′,3,3′-tetraethylbenzimidazolylcarbocyanine iodide) staining were not significantly different in comparison of WPE1-NA22 and WPE1-NB26 cells (Figures 3C,D). The data indicated that ATP levels were at least partially independent of mitochondrial respiration. These combined data suggest WPE1-NB26 cells may have an anaerobic glycolytic phenotype, a feature of highly aggressive cancer cells [21]. Metabolic switch from aerobic to anaerobic pathways concurrent with the progression of cancer also points to the possibility of less ROS production from mitochondrial respiration, which may result in a more reducing environment.

#### 2.2.2. Redox Characteristics of RPWE1, WPE1-NA22, or WPE1-NB26 Cells

**Figure 4.**
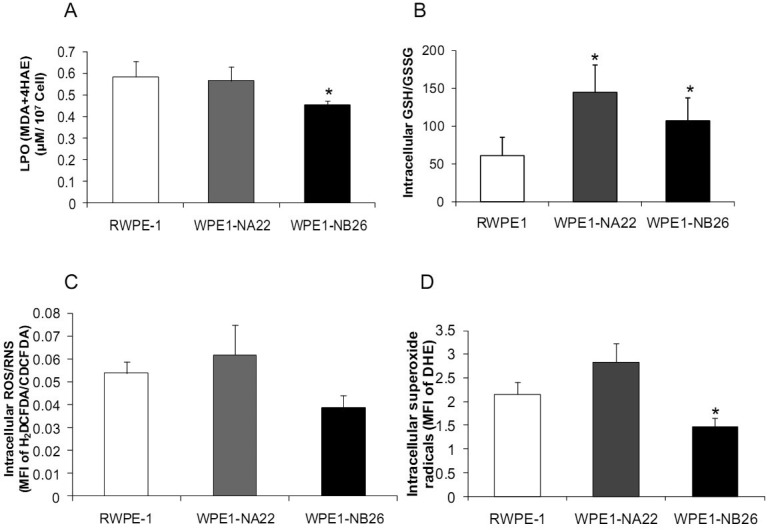
Intracellular redox state of RWPE1, WPE1-NA22, or WPE1-NB26 cells. (**A**) Lipid peroxidation levels. Cells were collected and cell lysates were measured for malondialdehyde (MDA) and 4-hydroxyalkenals (4HAE) levels using LPO-586 kit; (**B**) Intracellular GSH/GSSG ratios. Cells were cultured in KSFM without BPE and rEGF for 24 h. Cells were collected for GSH/GSSG assay; (**C**) Flow cytometry analysis of intracellular ROS/RNS levels. Cells were collected during log phase and incubated with 2′,7′-dichlorofluorescein diacetate (H_2_DCFDA). 5-(and-6)-carboxy-2′,7′dichlorofluorescein diacetate (CDCFDA, oxidation insensitive dye) was used to normalize uptake, efflux, and ester cleavage of H_2_DCFDA; (**D**) Flow cytometry analysis of intracellular superoxide radical levels. Cells (1 × 10^4^) were collected during log phase and incubated with dihydroethidium (DHE). MFI = Mean fluorescence intensity. * P-value ≤ 0.05 when compared to RWPE1.

Analyses of intra- and extracellular redox states were performed in RWPE1, WPE1-NA22, and WPE1-NB26 cells during log phase growth. As demonstrated in Figure 4, the most aggressive WPE1-NB26 cells showed the following statistically significant redox characteristics in comparison to the other two cell lines: decreased LPO, increased intracellular GSH/GSSG ratio (when compared with RWPE1 cells but not WPE1-NA22 cells), decreased intracellular ROS/RNS and O_2_^•−^ levels. It is possible that the aggressive nature of WPE1-NB26 cells may be related to the ability to grow in low levels/absence of a prooxidant state. These data correlated with previous findings that PC3 cells have very low levels of ROS/RNS and despite a low prooxidant state, proceed through the cell cycle [19].Elevation of extracellular H_2_O_2_ and nitrite levels in the media of WPE1-NB26 cells was previously demonstrated [20].

Since subcellular organelles within a particular cell type have an optimal redox state, it is likely that alteration of organelle redox state will result in cell adaptation or dysfunction. Antioxidant enzymes and antioxidant compounds are maintained at precise levels in cellular organelles both to maintain optimal subcellular redox state and to detoxify excess ROS/RNS. Cellular/subcellular redox state depends on levels of antioxidant compounds and proteins. Therefore, we measured levels of antioxidant and glutathione-related proteins. Various redox-associated proteins and GSH-related proteins in cytoplasm, mitochondria, and cell membrane were analyzed by western blotting using specific antibodies. Glyceraldehyde 3-phosphate dehydrogenase (GAPDH) was used as a protein loading control; initial studies demonstrated that GAPDH levels did not significantly vary between these cell lines.

**Figure 5.**
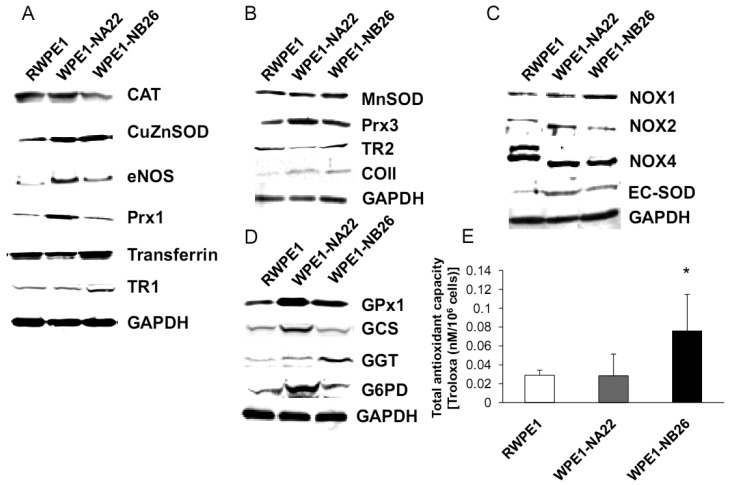
Western blot analysis of redox-related immunoreactive proteins of RWPE1, WPE1-NA22, or WPE1-NB26 cells. Cells were collected during log phase and cell lysates were used for analysis. (**A**) Cytoplasmic redox-related protein expression levels; CAT, CuZnSOD, eNOS, Prx1, TF, and TR1; (**B**) Mitochondrial redox-related protein expression levels; MnSOD, Prx3, TR2, and COII; (**C**) Cell membrane redox-related protein expression levels; NOX1, NOX2, NOX4, and EC-SOD; (**D**) Glutathione-related enzyme protein expression levels; GPx1, GCS, GGT, and G6PD. The figures are representative of three independent experiments; (**E**) Total antioxidant capacity. * P-value ≤ 0.05 when compared with RWPE1 cells.

As shown in Figure 5A, CuZnSOD, eNOS, and Prx1 protein levels were increased in WPE1-NA22 cells when compared to RWPE1 cells (P-value ≤ 0.08). High levels of specific antioxidant proteins in WPE1-NA22 cells could possibly be due to high levels of ROS/RNS or redox imbalance in the cells. CuZnSOD was also increased in WPE1-NB26 cells in comparison to RWPE1 cells (P-value ≤ 0.05). The levels of MnSOD, Prx3, and mitochondrial gene cytochrome oxidase subunit II (COII) protein, were slightly increased (P-value ≤ 0.1) in WPE1-NB26 cells in comparison to RWPE1 cells (Figure 5B). It is noteworthy that mitochondria are a major source of ROS production and an alteration in mitochondrial bioenergetics might result from changes in the mitochondrial antioxidant system. Some redox-related protein levels located on the cell membrane (EC-SOD and NOX1) were higher (P-value ≤ 0.05) in WPE1-NB26 than in RWPE1 cells (Figure 5C). These latter data correlated with induction of H_2_O_2_ levels in the media [20]. We postulate that higher extracellular ROS/RNS levels observed in WPE1-NB26 cells could be due to higher levels of NOX1. NOX2 and NOX4 isoforms demonstrated a shift in molecular weight in WPE1-NA22 and -NB26 in comparison to RWPE1 cells. Up-regulation of plasma membrane-bound NOX proteins has been demonstrated in several studies and may act as a potential source of increased ROS production [37]. Each cell line expressed different NOX isoforms and protein expression levels. For instance, a series of cell lines developed from the LNCaP cell line (designated as C4-2B cell lines) with increasing metastatic ability also showed increased NOX1 and H_2_O_2_ levels. Highly aggressive DU145 prostate cancer cells demonstrated elevation of NOX2 and p22^phox^ [37]. However, only a few studies have measured NOX enzyme activity, so whether or not the NOX family is actually a source of ROS in prostate cancer cells remains unclear [38]. Protein levels are not always equivalent to activity since proteins can be inactivated by ROS/RNS; thus, experiments to evaluate enzyme activity will be necessary to fully understand the significance of changes in protein levels.

Additional findings implicate imbalance of redox state in prostate cancer cell progression with alterations of the GSH-redox related antioxidant system, suggesting that these proteins are important for prostate cancer cell viability and survival under oxidative stress [19]. We found that levels of GSH-related proteins were significantly changed in WPE1-NA22 and WPE1-NB26 cells (Figure 5D). GPx1, GCS, and G6PD were significantly increased in WPE1-NA22 cells (P-value ≤ 0.05), whereas GPx1 and GGT were significantly increased in WPE1-NB26 cells when compared to RWPE1 cells (P-value ≤ 0.05). More importantly, total AC was significantly greater in WPE1-NB26 cells than RWPE1 or WPE1-NA22 cells (Figure 5E). GGT plays a key role in the gamma-glutamyl cycle, a pathway for the synthesis and degradation of GSH; thus, the higher levels of AC in WPE1-NB26 cells could possibly be due to a higher level of GGT protein expression. These data correlated with lower levels of intracellular LPO and ROS/RNS in WPE1-NB26 cells (Figures 4A–D). The intracellular redox characteristics of WPE1-NB26 cells were similar to the intracellular redox characteristics of highly aggressive human prostate PC3 cells. Thus, intra- and extracellular ROS/RNS and antioxidant redox-related proteins demonstrated distinct and similar redox state profiles in these two aggressive cell types.

#### 2.2.3. Effect of Intra- or Extracellular Redox States on RPWE1, WPE1-NA22, or WPE1-NB26 Cell Invasion

Alterations of redox states in prostate cancer cells may affect several functions in cancer cells, such as cell invasion and metastasis. The importance of extracellular redox state on WPE1-NB26 cell invasion has been previously demonstrated [20]; the extracellular redox state of WPE1-NB26 cells was more reducing (as analyzed by GSH/GSSG ratio and H_2_O_2_ levels) in comparison to RWPE1 cells. We also demonstrated that extracellular superoxide radical increased WPE1-NB26 cell invasion [20]. Using an adenoviral expression vector containing human EC-SOD, we showed that the invasive ability of WPE1-NB26 cells was reduced by overexpression of EC-SOD with decrease of MMP2 and MT1-MMP enzymatic activities correlating with decrease in invasiveness [20]. These results suggest that increased ROS, perhaps at the cell surface, in the more aggressive cells are at least partially responsible for increased invasive ability of these cells, and invasion may be at least partially regulated by MMP. Thus, modulation of plasma membrane/extracellular redox state resulted in alterations in prostate cancer cell behavior.

**Figure 6.**
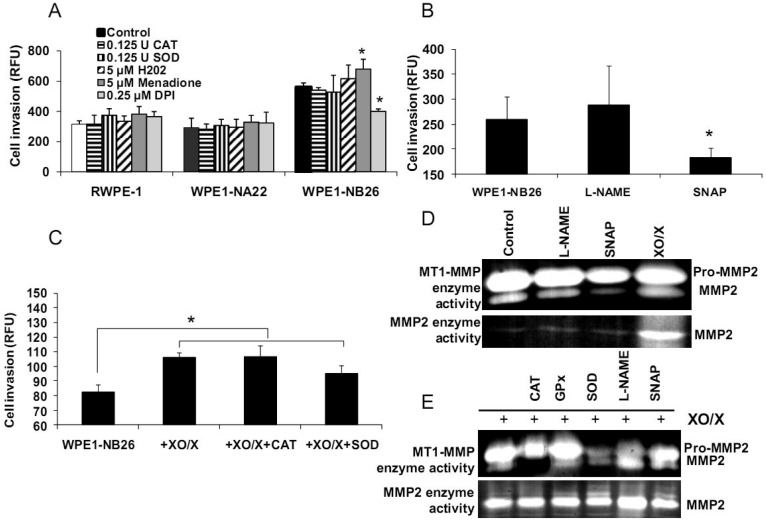
Effects of antioxidant enzymes and low molecular weight redox modulators on cell invasion and MMP activities of WPE1-NB26 cells. (**A**) RWPE1, WPE1-NA22, or WPE1-NB26 cells were incubated with 0.125 U of CAT, 0.125 U of SOD, 5 μM H_2_O_2_, 5 μM menadione, or 0.25 μM DPI during cell invasion assays for 24 h; (**B**) WPE1-NB26 cells were incubated with 100 μM SNAP or 100 μM L-NAME for 24 h during cell invasion assays; (**C**) WPE1-NB26 cells were incubated with 100 μM XO/X ± CAT or 100 μM XO/X ± SOD during cell invasion assay; (**D**) MMP2 and MT1-MMP zymographies. WPE1-NB26 cells were cultured in KSFM (without BPE and rEGF) containing 100 μM XO/X, 100 μM SNAP, or 100 μM L-NAME for 24 h; (**E**) MMP2 and MT1-MMP zymographies. WPE1-NB26 cells were cultured in KSFM (without BPE and rEGF) containing 100 μM XO/X and co-incubated with CAT, GPx, SOD, L-NAME, or SNAP for 24 h. Conditioned media were collected and analyzed for MMP2 activity by zymography. Gelatinolytic activities of MMP2 were detected as clear bands against a blue background. For MT1-MMP zymography, 20 ng of pro-MMP2 were added in the medium before co-incubation with redox-modulating compounds. The ability of MT1-MMP to catalyze pro-MMP2 (upper bands) to MMP2 (lower bands) was detected. RFU = Relative fluorescence units. * P-value ≤ 0.05 when compared with control.

We demonstrated differences of antioxidant and ROS/RNS levels among these cell lines; thus, intracellular ROS/RNS may play a role in prostate cancer cell invasion. To further test this hypothesis, RWPE1, WPE1-NA22, or WPE1-NB26 cells were treated with 0.125 Unit (U) CAT, 0.125 U SOD, 5 μM H_2_O_2_, 0.25 μM DPI, 5 μM menadione (O_2_^•−^ generator), 100 μM NG-nitro-L-arginine methyl ester (L -NAME, nitric oxide inhibitor), 100 μM S-nitroso-N-acetylpenicillamine (SNAP; a ^•^NO donor), or xanthine oxidase (XO)/xanthine (X) (an O_2_^•−^ generating system) for 24 h, and *in vitro* cell invasion assays were performed. As shown in Figures 6A-B, menadione treatment significantly increased WPE1-NB26 cell invasion, whereas DPI and SNAP treatment significantly decreased WPE1-NB26 cell invasion. These changes were specific to WPE1-NB26 cells since they were not observed in RWPE1 or WPE1-NA22 cells (Figure 6A). In addition, MMP2 and MT1-MMP activities of WPE1-NB26 cells were significantly decreased after treatment with SNAP, whereas MMP2 activity was significantly increased after treatment with XO/X. We conclude that intracellular redox state regulated invasion of highly aggressive prostate epithelial WPE1-NB26 cells, with O_2_^•−^ acting as an enhancer and ^•^NO acting as an inhibitor. We performed further analysis by incubation of WPE1-NB26 cells with XO/X ± CAT or SOD for 24 h before invasion assays were performed. As expected (Figure 6C), XO/X treatment significantly increased cell invasion, simultaneous treatment of XO/X with CAT did not change cell invasion whereas simultaneous treatment of XO/X with SOD slightly decreased cell invasion. Treatment with CAT or SOD significantly decreased MT1-MMP activity during XO/X treatment (Figure 6E). Thus, superoxide radical is a key molecule for induction of WPE1-NB26 cell invasion, at least partially through activation of MMP2 or MT1-MMP. These data support the important role of extra-and/or intracellular redox states in aggressive prostate cancer cell invasion, since cell invasion in RWPE1 or WPE1-NA22 cells did not change when treated with the same conditions (Figure 6A).

Our laboratory has documented an important finding that aggressive prostate cancer cells showed elevated levels and altered subcellular distribution of antioxidant proteins in comparison with primary prostate cancer [18,39], suggesting the possibility of manipulation of cell redox state for successful treatment of the metastatic process. These data correlated with the results found in PC3 cells. In addition, cross talk between intra- and extracellular redox states might play a critical role in cancer migration and invasion.

#### 2.2.4. Effects of TNF, TRAIL, or β-Phenethyl Isothiocyanate (PEITC) on RPWE1, WPE1-NA22, or WPE1-NB26 Cell Viability

**Figure 7.**
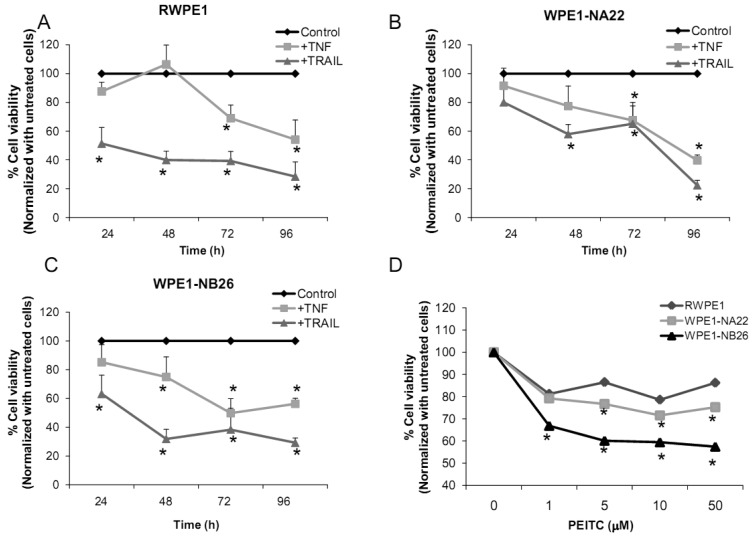
Effects of TNF, TRAIL, or PEITC on cell viability of RWPE1, WPE1-NA22, or WPE1-NB26 cells. (**A**) RWPE1; (**B**) WPE1-NA22, or (**C**) WPE1-NB26 cells were treated with 40 ng/mL TNF or TRAIL for 24, 48, 72, and 96 h, or (**D**) RWPE1, WPE1-NA22, and -NB26 cells treatment with PEITC at different concentrations (0, 1, 5, 10, and 50 μM) for 24 h. MTT assays were performed. * P-value ≤ 0.05 when compared with control.

Several redox-related compounds have been used as cancer therapeutic agents. However, differences in redox characteristics such as antioxidant levels of each individual cancer may determine the success of the treatment. The molecules chosen for analysis were TNF or TRAIL since these molecules have previously demonstrated the ability to kill PC3 cells. As shown in Figures 7A-C, TNF treatment (40 ng/mL) at 48 h significantly inhibited cell viability of WPE1-NA22 or WPE1-NB26 cells when compared to RWPE1 cells. At 72 and 96 h, TNF treatment significantly inhibited cell viability of all cell lines. TRAIL (40 ng/mL) treatment significantly inhibited cell viability in all three cell lines. These studies strongly supported an important relationship between redox state and cancer and correlated with PC3 cell data that TNF induced cell death in highly aggressive prostate cancer cells. Induction of MnSOD protein expression levels was also observed in WPE1-NB26 cells treated with TNF (data not shown). Induction of MnSOD may induce H_2_O_2_ mediated cell death. A previous study has reported the importance of intracellular ROS/RNS for growth of normal and cancer cells [40]. Among the reactive species, H_2_O_2_ and ^•^NO have been conclusively documented to be important intracellular messengers. H_2_O_2_ is produced in response to the binding of many growth factors with their receptors, examples being epidermal growth factor, TNF, insulin, and cytokines [29,30,31]. It has been suggested that these cell surface receptors might produce H_2_O_2_ by activating NADPH oxidase isoforms with resultant cell surface H_2_O_2_ traveling through plasma membrane to the cytosol and contributing to intracellular signaling pathways [30]. In addition, H_2_O_2_ can further oxidize cysteine residues in proteins, such as protein tyrosine phosphatases and protein kinases, to regulate diverse cellular processes, including cell death, cell migration, and cell proliferation [29,31].

We analyzed the sensitivity of prostate cancer cells to PEITC (GSH inhibitor), a chemical mostly found in cruciferous vegetables which is produced upon cutting or chewing of these vegetables [41]. We found that PEITC significantly inhibited WPE1-NB26 cell viability after 24 h treatment, whereas RWPE1 or WPE1-NA22 cells were less sensitive to PEITC (Figure 7D). PEITC-mediated cell death is dependent on two important factors: the biological differences in redox regulation between oncogenically transformed cells and normal cells, and the ability of PEITC to effectively abolish the glutathione-dependent antioxidant system [26,42,43]. WPE1-NB26 cells demonstrated lower levels of intracellular ROS/RNS and higher levels of intra- and extracellular GSH/GSSG ratios. Therefore, glutathione-related proteins are most likely important for WPE1-NB26 cell survival under oxidative stress conditions. Moreover, similar results were found in PEITC treated PC3 cells, which indicated that PEITC selectively induced cell death in PC3 but not PrEC cells [26]. PEITC treatment resulted in generation of ROS production (possibly H_2_O_2_) in PC3, but not in PrEC cells [26]. The differences in basal antioxidant defense mechanisms and redox state between PC3 *versus* PrEC cells and RWPE1 *versus* WPE1-NB26 cells were mostly likely responsible for differential sensitivity to prooxidant effects of PEITC. While this susceptibility may have important therapeutic implications for aggressive prostate cancer, future studies will be necessary to document efficacy *in vivo*.

### 2.3. Androgen-Responsive LNCaP and Highly Aggressive LNCaP-C4-2 Prostate Cancer Cells

WPE1-NA22 and -NB26 cells are a prostate cancer progression model that derived from the same cell type (RWPE1 cells). However, this model does not truly represent aggressive human prostate cancer since RWPE1 did not originate from human prostate cancer. To further confirm our hypothesis of the importance of cell redox state in human cancer progression, androgen-responsive LNCaP and LNCaP-C4-2 cells were selected for further study. The LNCaP cell line was established from a biopsy of a lymph node metastasis from a 50-year-old Caucasian [44]. A series of lineage-related LNCaP cell sublines that reflect the various steps of prostate carcinogenesis and progression has been derived [45,46]. LNCaP-C4-2 cells were derived by co-culturing LNCaP cells with a human bone fibroblast cell line (MS) established from a patient with an osteogenic sarcoma. LNCaP tumors were induced by subcutaneous co-injection of LNCaP cells (1 × 10^6^) and MS cells (1 × 10^6^) into nude athymic mice. After 8 weeks, mice were castrated. After an additional 4–5 weeks, tumors were harvested, and the LNCaP-C4-2 cell line was established. Thus, LNCaP and LNCaP-C4-2 cell lines have varying degrees of aggressiveness and are derived from a cancer patient.

#### 2.3.1. Analysis of Growth, Invasion, and Antioxidant Protein Expression Levels of LNCaP and LNCaP-C4-2 Cell Lines

**Figure 8.**
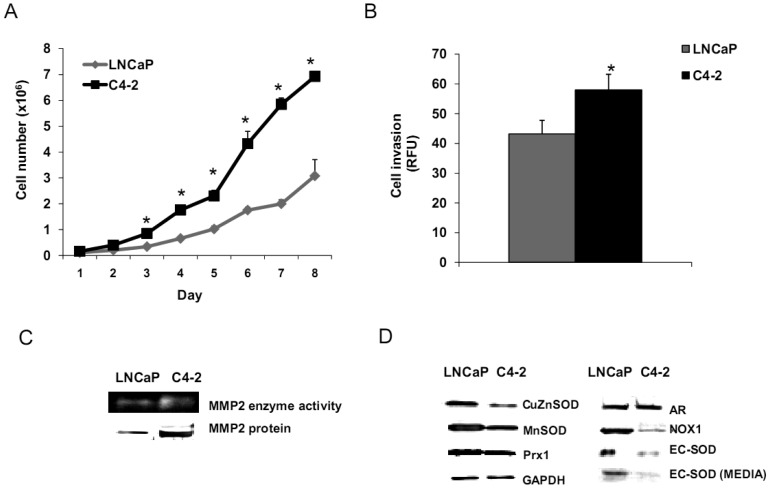
Biology and redox related protein expression of LNCaP or LNCaP-C4-2 cells. (**A**) Growth curves. Cells were seeded at 7 × 10^4^ cells in 60 mm dishes and counted every 24 h; (**B**) Invasion ability and (**C**) MMP activity (zymography) and protein level (western blot analysis). Cells were cultured for 24 h in RPMI medium without serum prior to analysis. Cells were collected for invasion assay and conditioned media were collected for MMP2 zymography. For invasion assay, after incubation for 24 h, cells that had invaded through Matrigel matrix membrane-coated polycarbonated (PCF) inserts were detached and counted using Calcein-AM fluorescence dye; (**D**) Western blot analysis of redox-related immunoreactive proteins. Cells or conditioned media were collected during log phase and were used for analysis of CuZnSOD, MnSOD, Prx1, androgen receptor (AR), NOX1, or EC-SOD. * P-value ≤ 0.05 when compared to LNCaP cells. C4-2 = LNCaP-C4-2 cells.

We analyzed biologic behavior and redox biochemistry of the less aggressive LNCaP prostate carcinoma cell line in comparison with the more aggressive LNCaP-C4-2 prostate carcinoma cell line. These initial tissue culture studies were performed in the absence of androgen. As shown in Figure 8A, the results demonstrated that LNCaP-C4-2 cells grew faster than LNCaP cells. LNCaP-C4-2 cells had greater invasion ability through Matrigel matrix basement membrane when compared with LNCaP cells (Figure 8B), correlating with higher levels of MMP2 protein and activity (Figure 8C). To better understand redox differences between these two cell lines, various redox-associated proteins were analyzed by western blotting using specific antibodies. Western blot analysis of redox-related proteins indicated that LNCaP cells had greater levels of certain antioxidant proteins; CuZnSOD, MnSOD, and Prx1 (Figure 8D). Furthermore, total SOD, MnSOD, and EC-SOD enzymatic activities in LNCaP-C4-2 cells were lower than LNCaP cells (data not shown). Lower levels of MnSOD protein were often found in malignant tissues of primary cancers compared to corresponding non-tumor tissues [18,39]. As mentioned earlier, SODs have dual effects as antioxidant and prooxidant; lower levels of SODs in more aggressive prostate cancer cell lines may be a compensatory mechanism to lower oxidative stress levels due to the prooxidant effect of SODs.

**Figure 9.**
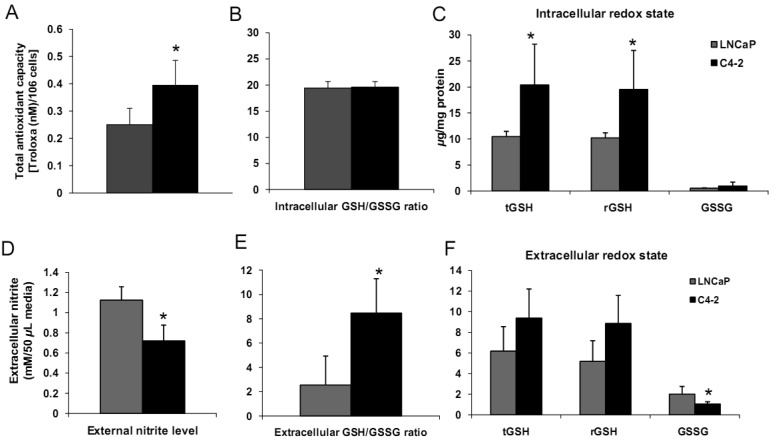
Analysis of redox states in LNCaP *vs.* LNCaP-C4-2 cells. Cells were cultured in RPMI without serum for 24 h before cells and conditioned media were collected for further analysis. (**A**) Total antioxidant capacity; (**B**) Intracellular GSH/GSSG ratio; (**C**) Intracellular GSH and GSSG levels; (**D**) Extracellular nitrite levels. Nitrite levels in conditioned media were analyzed based on the Griess reagent system; (**E**) Extracellular GSH/GSSG ratio; (**F**) Extracellular GSH and GSSG levels. * P-value ≤ 0.05 when compared with LNCaP cells. tGSH = total GSH, rGSH = reduced GSH, C4-2 = LNCaP-C4-2 cells.

Levels of cell membrane redox-related proteins NOX1 and EC-SOD were significantly higher in LNCaP cells compared to LNCaP-C4-2 cells, where as AR expression levels of LNCaP and LNCaP-C4-2 cells were not significantly different (Figure 8D). In contrast, total AC and total GSH of LNCaP-C4-2 cells were significantly higher than LNCaP cells (Figure 9A). The reverse correlation of antioxidant protein expression and total AC could possible due to post-translational processes in LNCaP cells or intracellular redox imbalance with resultant inhibition of AE activity. As shown in Figures 9C–F, LNCaP cells had higher levels of extracellular nitrite whereas LNCaP-C4-2 cells had a higher extracellular GSH/GSSG ratio, indicating a more reduced microenvironment in LNCaP-C4-2 cells. It is not certain if the induction of AC and increase in extracellular GSH/GSSG ratios is due to slight increase of intracellular ROS/RNS levels inside LNCaP-C4-2 cells (data not shown). An alternative/additional explanation of our results is that more aggressive LNCaP-C4-2 cells maintain low levels of oxidative stress via an altered or more extensive antioxidant network.

The levels of antioxidant parameters were consistently higher in more aggressive cancer cells (PC3, WPE1-NB26, and LNCaP-C4-2 cells) than less aggressive cancer cells (WPE1-NA22 and LNCaP cells). These findings are correlated with the expression of the transcription factor, nuclear factor-erythroid-related factor 2 (Nrf2). Nrf2 is a redox-regulated transcription factor that regulates several antioxidant proteins including TR1, Prx1, and GST. Western blot analysis in PrEC, LNCaP, PC3, and DU145 cell lines showed that Nrf2 downstream target antioxidant proteins; TR1, TR2, Prx1, and quinone oxidoreductase were overexpressed in LNCaP, PC3, and DU145 cells when compared with PrEC cells (data not shown). These data correlated with recent studies by DeNiola *et al.* [47]; they found that activation of Nrf2 by dominant oncogenes with resultant ROS-detoxification contributes to tumorigenesis in mouse embryonic and NIH3T3 fibroblasts. Our data and theirs indicate greater redox buffering capacity in highly aggressive prostate cancer, which may be used as an adaptive mechanism to keep excess ROS within ranges that permit cancer cells to grow and survive. Prostate cancer progression may therefore critically depend on the synergy between ROS/RNS and antioxidant levels [48].

#### 2.3.2. Effects of Androgen on Cell Growth in LNCaP or LNCaP-C4-2 Cell Lines

**Figure 10.**
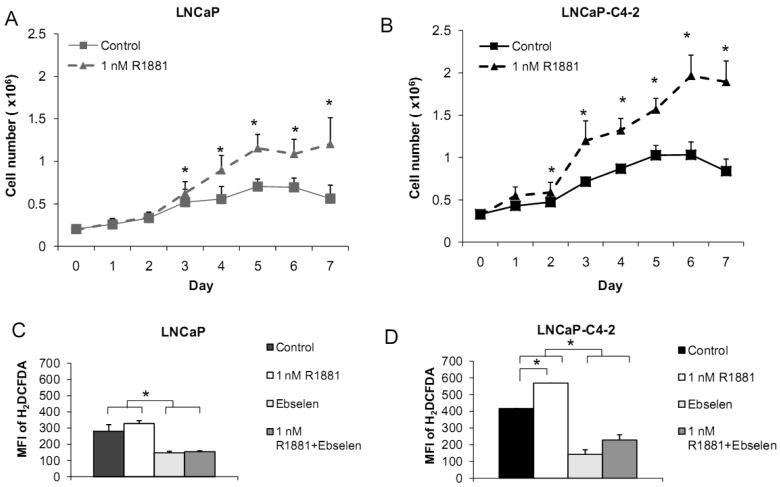
Effects of an androgen analog R1881 on LNCaP or LNCaP-C4-2 cell growth and ROS/RNS levels. (**A**) LNCaP cells or (**B**) LNCaP-C4-2 cells were cultured with 1 nM R1881 for 7 days. Cell numbers were counted every 24 h; (**C** and **D**) Flow cytometry analysis of intracellular ROS/RNS levels after LNCaP or LNCaP-C4-2 cells were treated with 1 nM R1881 ± 25 μM Ebselen for 48 h. Cells (1 × 10^4^) were collected during log phase and incubated with H_2_DCFDA. CDCFDA (oxidation insensitive dye) was used to normalize uptake, efflux, and ester cleavage of H_2_DCFDA. Data are presented as MFI of H_2_DCFDA; MFI of CDCFDA of LNCaP and LNCaP-C4-2 cells were not significantly different. * P-value ≤ 0.05 when compared to control.

Based on the demonstrated unique redox properties of LNCaP and LNCaP-C4-2 cells and their responsiveness to androgen, we studied the effects of R1881 (Methyltrienolone, a synthetic androgen) on cell growth. Cells were treated with 1 nM of R1881 for seven days. As shown in Figures 10A,B, R1881 treatment significantly stimulated growth of both LNCaP and LNCaP-C4-2 cells. Cell growth was significantly less in LNCaP cells. AR protein was found to be expressed by these two cell lines at comparable levels. AR in LNCaP-C4-2 cells is highly active and the co-operative binding of AR to the multiple androgen response elements (AREs) on the enhancer core allows the core to be transcriptionally active even in the absence of androgen. Thus, activated AR in LNCaP-C4-2 cells might give them growth advantages over LNCaP cells in an androgen-deprived condition. We measured intracellular ROS/RNS levels during 48 h treatment with R1881 using H_2_DCFDA with flow cytometry. As demonstrated in Figures 10C-D, intracellular ROS/RSN levels were significantly higher in LNCaP-C4-2 cells than LNCaP cells. Treatment of R1881 significantly increased the levels of intracellular ROS/RNS in LNCaP-C4-2 cells and slightly but not significantly in LNCaP cells. In addition, co-incubation with Ebselen (GPx mimic) demonstrated a significant decrease in intracellular ROS/RNS levels in both cell types. These results indicated induction of ROS/RNS by R1881 treatment. We previously demonstrated a strong correlation between LNCaP cell cycle progression and levels of ROS/RNS [19]; thus, elevation of ROS/RNS levels after R1881 treatment possibly induced cell growth in both LNCaP and LNCaP-C4-2 cells. Induction of ROS/RNS was at least partially due to increased mitochondrial ROS levels and mitochondrial redox regulation of p66^Shc^ [49,50]. An additional study indicated that the transcription factor JunD is an essential mediator of androgen-induced ROS levels [51]. A study in our laboratory demonstrated a transient cytoplasmic oxidation of Trx1 after androgen treatment, indicating a possible involvement of androgen in redox signaling [52]. Thus, a shift in prooxidant-antioxidant balance toward the prooxidant state was observed in aggressive prostate cancer cells after treatment with R1881.

**Figure 11.**
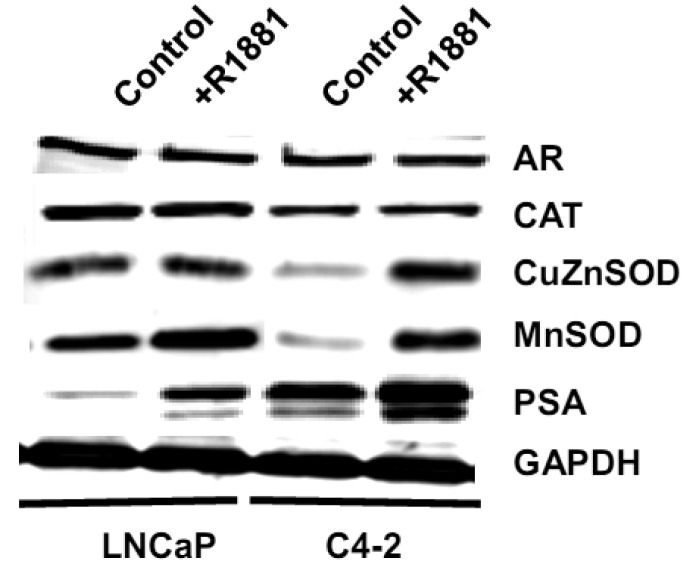
Effect of androgen on levels of redox-related protein expression in LNCaP and LNCaP-C4-2 cells. Cells were treated with 1 nM R1881 for 48 h. Cells were collected and lysates were used for western blot analysis. AR, CAT, CuZnSOD, MnSOD, and PSA immunoreactive protein levels were analyzed. C4-2 = LNCaP-C4-2 cells.

We measured levels of redox-related protein expression after R1881 treatment (Figure 11) and found that MnSOD and CuZnSOD protein expression was increased in LNCaP-C4-2 cells, whereas only MnSOD was increased in LNCaP cells. Since MnSOD and CuZnSOD have dual effects as antioxidant and prooxidant [40], induction of these proteins could possibly be due to an elevation of ROS/RNS. On the other hand, elevation of ROS/RNS after R1881 treatment could be due to induction of MnSOD and CuZnSOD. A shift in prooxidant state by R1881 is also supported by other studies which demonstrated induction of CAT activity and LPO and decrease in GSH [50]. These changes seen after R1881 treatment were likely due to a cellular response to increased production of ROS/RNS. Prostatic specific antigen (PSA) levels were significantly increased in LNCaP cells and slightly increased in LNCaP-C4-2 cells after R1881 treatment (Figure 11). PSA levels were higher in LNCaP-C4-2 cells than LNCaP cells without treatment with androgen, most likely due to constitutive activation of AR in LNCaP-C4-2 cells. Sharifi *et al.* examined the role of MnSOD in the function of the AR using MnSOD siRNA and found that MnSOD knockout resulted in up-regulation of androgen-regulated gene expression and induction of androgen-DNA binding [53]. Changing redox balance after treatment with R1881 may act as a growth regulator or a growth inhibitor in androgen-responsive prostate cancer cells depending on the cell redox state and concentration of R1881 [50].

## 3. Experimental Section

### 3.1. Chemicals and Reagents

All chemicals and reagents were purchased from Sigma-Aldrich Chemical Co. (St. Louis, MO, USA), unless otherwise specified. Tissue culture supplies were from Falcon (Becton-Dickinson Labware, Franklin Lakes, NJ, USA). All tissue culture reagents were obtained from Invitrogen Life Technologies (Carlsbad, CA, USA), except for fetal bovine serum (FBS) which was purchased from Tissue Culture Biological (Tulare, CA, USA). Amplex Red Hydrogen peroxide/Peroxidase assay kit, ATP assay kit, CDCFDA, H_2_DCFDA, DHE, Hoechst 33842, and JC-1 were obtained from Invitrogen Life Technologies. R1881 was purchased from PerkinElmer (Waltham, MA, USA). Total antioxidant assay kit was purchased from Sigma-Aldrich Chemical Co. Griess reagent system was purchased from Promega Co. (Madison, WI, USA). PCF inserts were purchased from Millipore Co. (Billerica, MA, USA). Pro-MMP2 and calcein-AM fluorescence dye were purchased from Merck Chemicals Ltd. (Darmstadt, Germany). Matrigel matrix membrane was purchased from BD Biosciences (Bedford, MA, USA).

### 3.2. Cell Culture

RWPE1, WPE1-NA22, WPE1-NB26, LNCaP and PC3 cell lines were obtained from ATCC (Manassas, VA, USA). LNCaP and PC3 cells were tested and confirmed for authenticity using short tandem repeats DNA typing by Biosynthesis Cell Inc. (Lewisville, TX, USA). LNCaP-C4-2 was obtained from ViroMed Laboratories (Minnetonka, MN, USA). PrEC and PrEGM were obtained from Lonza Walkersville Inc. (Walkersville, MD, USA). LNCaP, LNCaP-C4-2, and PC3 cells were cultured in RPMI 1640 medium supplemented with 5% serum and 100 mg/L kanamycin sulfate. RWPE1, WPE1-NA22, and WPE1-NB26 cells were cultured in KSFM supplemented with 50 mg/L BPE, 5 μg/L rEGF, and 100 mg/L kanamycin sulfate. PrEC cells were cultured in PrEGM medium. For androgen treatment experiments, cells were cultured in RPMI 1640 medium supplemented with 5% charcoal stripped FBS. All cells were grown at 37 °C in a humidified atmosphere of 95% air and 5% CO_2_. Trypsin (0.05%) and 0.53 mM EDTA were used for routine subculture. Cells passaged less than 25 times were used in all experiments. Mycoplasma contamination was tested using the Mycosensor PCR Assay Kit (Stratagene, La Jolla, CA) and was negative in the cultures used for these studies. Catalase, Ebselen, DPI, H_2_O_2_, L-NAME, menadione, PEITC, R1881, SNAP, SOD, TNF, TRAIL, or XO/X were added to the media at various concentrations and time points, then cells and/or conditioned media were collected for further analysis. For each cell line, preliminary time-course and dose-response studies of these compounds were performed, with only the optimal conditions resulting in a biologic response reported in the present study.

### 3.3. Cell Viability Assay

Cells were plated at 5 × 10^5^ cells per well using 24-well plates. Cells during exponential phase were then treated with 200 MOI of Adhsod2, 1–50 μM PEITC, 1 nM R1881, 40 ng/mL TNF, or 40 ng/mL TRAIL for various times. Cells were incubated with MTT solution for 2 h at 37 °C in 5% CO_2_/95% air and then dissolved in acidified isopropanol. Optical density was detected at 570 nm using a spectrophotometer (DUSeries 640, Beckman Coulter Inc., Fullerton, CA, USA).

### 3.4. Extracellular Nitrite Measurement

External nitrite levels (which are a stable and nonvolatile breakdown product of ^•^NO) were analyzed following the manufacturer's instructions based on the Griess reagent system as previously described [20]. Media decanted from growing cells were analyzed in these experiments.

### 3.5. Cell Growth Curve

Cells were seeded at 7 × 10^4^ cells per 60 mm dish. To insure adequate cell recovery following the addition of trypsin plus EDTA, cell counting was initiated at 48 h after plating. Media were renewed on days 1, 3, and every day during the log phase of growth; this protocol was adopted to prevent large pH shifts during the most rapid phase of growth. Cells were trypsinized every 24 h and counted using a particle counter, Coulter Z1 series (Beckman Coulter Inc.).

### 3.6. Glutathione Assay

Cell lysates or conditioned media were collected for intra- or extracellular glutathione assays (total GSH, reduced GSH, and GSSG levels), respectively. All the samples were protected from light and exposure to air for the minimal but the same amount of time. Sample preparations and assay protocol based on 5,5′-dithiobis-(2-nitrobenzoic acid)-GSSG reductase recycling were previously described [19]. The GSH/GSSG ratio was calculated as an indicator of redox balance.

### 3.7. In Vitro Invasion Assay

Cells (7.5 × 10^5^/mL) were cultured in serum-free RPMI 1640 or KSFM with no BPE/rEGF added before seeding in the upper chamber. Serum-free RPMI 1640 or KSFM with no BPE/rEGF or complete media (plus FBS or plus BPE/rEGF) were placed in the upper and lower chambers, respectively. After incubation for 24 h with or without redox-modulating compounds at 37 °C in 5% CO_2_/95% air, cells that had invaded through coated PCF membranes were detached using the cell dissociation buffer and detected by the calcein-AM fluorescence dye. Collagen type I-coated (1 mg/mL) and Matrigel matrix-coated (100 μg/mL) PCF membranes were used for RWPE1 family and LNCaP/LNCaP-C4-2 cell lines, respectively.

### 3.8. Intracellular ROS/RNS Measurement

For analysis of steady state intracellular ROS/RNS levels, cells at log phase were collected using 1 mM EDTA, and 5 × 10^5^ cells/mL were incubated in RPMI 1640 (without phenol red and FBS) containing 10 μM H_2_DCFDA or CDCFDA for 30 min at 37 °C with light protection. CDCFDA (oxidation insensitive dye) at 10 μM was used to normalize uptake, efflux, and ester cleavage of H_2_DCFDA [21]. The experiments were performed on three consecutive days, using the same lot of fluorescence dyes; dyes were stored at −20°C and protected from light at all times. SPHERO Ultra Rainbow Calibration Particles (Spherotech Inc., Libertyville, IL, USA) were used as control for changes in photomultiplier gain on the flow cytometer each day. Cells (1 × 10^4^) were collected for analysis of the geometric mean fluorescence in living cells using a BD FACSCalibur flow cytometer (BD Biosciences, San Jose, CA, USA) equipped with FlowJo 6.1 software (TreeStar, Ashland, OR, USA). The data are presented as the ratio of MFI from each dye of 10,000 cells to MFI of 10,000 unstained cells. 25 μM Ebselen or 10 mM H_2_O_2_ were used as negative and positive controls, respectively.

### 3.9. ATP Assay

Cells (1 × 10^4^) during log phase were collected and ATP concentrations in cell homogenates were measured by using the ATP determination kit from Invitrogen Life Technologies. The assay is based on luciferase's requirement for ATP in producing light (emission maximum ∼560 nm at pH 7.8). Luminescence was read on a Synergy 4 microplate reader and values were calculated based on an ATP standard curve.

### 3.10. Mitochondrial Membrane Potential Assay

JC-1 fluorescence dye was used to determine the electrochemical gradient across the mitochondrial membrane (mitochondrial membrane potential). Cells (5 × 10^5^/mL) were collected and incubated with 10 μM Hoechst 33342 for 1 h at 37 °C. After incubation, cells were washed and incubated with 10 μM JC-1 fluorescence dye for 30 min at 37 °C with light protection. Cells were then analyzed by a BD FACSCalibur flow cytometer. JC1 fluorescence excited at 488 nm was detected with FL1 (green) and FL2 (red) channels.

### 3.11. Adenovirus Transduction

Adenoviral vectors containing human MnSOD cDNA or without cDNA insert were purchased from ViraQuest Inc. (North Liberty, IA, USA). An adenoviral construct containing green fluorescence protein cDNA (ViraQuest Inc.) was used to determine transduction efficiency. The adenovirus constructs used were described previously [22]. The procedure of transduction of adenoviral vectors was detailed elsewhere [20]. Briefly, cells were seeded (5 × 10^5^ cells/100 mm dish), allowed to attach for 48 h and were transduced with either Adhsod2 or AdEmpty at a specified MOI. At the end of the 24 h transduction period, fresh media were added and cells were cultured and MTT assays were performed at 24, 48, 72, and 96 h after transduction.

### 3.12. Lipid Peroxidation Measurement

Cells (1 × 10^7^) were collected during log phase and suspended in 5 mM butylated hydroxytoluene. Cells were then frozen and thawed for three cycles. Cell lysates were collected and measured for protein concentration after centrifugation at 3430 × g for 10 min at 4 °C. The assay was performed according to the manufacturer's instructions (LPO-586, OxisResearch, Portland, OR, USA); this assay measures MDA and 4HAE. Briefly, MDA and 4HAE reacts with *N*-methyl-2-phenylindole at 45 °C to yield a stable compound that can be measured at 585 nm using a DUSeries 640 spectrophotometer. Unknown quantities of LPO were determined by comparison with a 4HAE and MDA linear standard curve.

### 3.13. MMP Zymography

Conditioned media were concentrated using an Amicon Ultra-15 filter (Millipore Co.). Thirty micrograms of protein obtained from concentrated conditioned media were subjected to electrophoresis (12% SDS-PAGE copolymerized with 1% gelatin as substrate). Gels were then washed with 2.5% Triton X-100 and incubated at 37 °C overnight with Tris-HCl buffer (pH 7.6). Gels were stained with 0.25% Coomassie blue G-250 (Bio-Rad) for 1 h and destained with 10% acetic acid and 40% methanol until gelatinolytic activities were detected as clear bands against a blue background. The ability of MT1-MMP to catalyze pro-MMP2 to active MMP2 was analyzed by adding 20 ng of exogenous pro-MMP2 to media for 24 h. Conditioned media were collected, concentrated, and subjected to electrophoresis.

### 3.14. Total Antioxidant Capacity Assay

Cells (1 × 10^6^) were collected and sonicated on ice. Supernatants were used for measurement of total antioxidant capacity based on the manufacturer's protocol. The principle of the antioxidant assay is formation of a ferryl myoglobin radical from metmyoglobin and hydrogen peroxide, which oxidizes ABTS (2,2′-azino-bis(3-ethylbenzthiazoline-6-sulfonic acid) to produce a radical cation, ABTS^•+^, a soluble chromogen that is green in color and can be measured spectrophotometrically at 405 nm. Troloxa, a water-soluble vitamin E analog, was used to construct a standard curve of antioxidant capacity.

### 3.15. Western Blotting Analysis

The protocols of western blot analysis were detailed elsewhere [19]. Protein levels of all samples were determined by the Bradford assay according to manufacturer's instructions (Bio-Rad Laboratories, Hercules, CA, USA). Crude supernatants or concentrated conditioned media were placed in each well. The following primary antibodies were used: anti-CAT (1:1,000) from Athens Research and Technology (Athens, GA, USA), anti-CuZnSOD (1:1,000) from Dr. Larry Oberley's laboratory (Iowa City, IA, USA), anti-eNOS (4 μg/mL), anti-EC-SOD (1 μg/mL), MnSOD (1:1,000), and TF (1:1,000) from Stressgen Bioreagents (Victoria, BC, Canada), anti-GPx1 (1:1,000), anti-Prx1 (1:1,000) and 3 (1:1,000), anti-TR1 (1:1,000) and 2 (1:1,000) from Abfrontier (Seadaemun-gu, Seoul, Korea), anti- GCS (1:1,000), anti- GGT (1:200), anti-PSA (1:200) from Abcam Inc. (Cambridge, MA, USA), anti-G6PD (1:1,000) from Bethyl Laboratories Inc. (Montgomery, TX, USA), anti-AR, anti-COII, and anti-NOX1, 2, 3 (1:200) from Santa Cruz Biotechnology Inc. (San Diego, CA, USA). Anti-GAPDH (1:5,000; Abcam Inc.) antibody was used as protein loading control. Horseradish peroxidase-conjugated secondary antibodies (polyclonal antibody 1:10,000 and monoclonal antibody 1:5,000) (Santa Cruz) were used for the assay.

### 3.16. Statistics

Each experiment was performed at least three times. Statistical analysis was performed with student's *t* test or multiple comparisons were performed with one way ANOVA followed by multiple comparison tests using SPSS10 software (SPSS Inc., Chicago, IL, USA). Mean differences were considered significant at P-value ≤ 0.05 unless otherwise specified. All data were represented as Mean ± SEM.

## 4. Conclusions

Several studies have strongly emphasized the possible relationship of redox state to cancer. Several cancer cell lines have modulated levels of ROS/RNS compared to nonmalignant cells. Redox state has been implicated in tumor prevention; for example, in experimental models of skin cancer, antioxidants prevent tumorigenesis, and antioxidants are in clinical trials for prevention of human oral and prostate cancer. Agents known to generate ROS, including ionizing radiation and adriamycin, are cornerstones of cancer therapy. Therefore, studies of AEs and redox state of the cells have tremendous therapeutic potential for subsequent applications in the battle against cancer. The role of cell redox state and oxidative stress in prostate cancer development and progression is currently unclear. The present study partially analyzed and characterized the effects of cellular/subcellular redox state on prostate cancer cell viability, growth, and invasion *in vitro*. We characterized selected prostate cancer cell lines that represent different progression stages in human cancer and found that each stage displayed distinct redox characteristic profiles as summarized in Table 1.

This finding may have diagnostic use in future studies in distinguishing nonmalignant from malignant disease and result in new cancer therapies based on differences in redox state biochemistry. As an example, if the highly metastatic phenotype is at least partially due to altered levels of AEs, then therapy designed to modify the levels of AEs might reduce cell proliferation and cell invasiveness in highly metastatic lesions and make the cancer more amenable to adjuvant therapy. Knowledge of subcellular redox profiles can help to predict the response of each cell type to selected low-molecular-weight redox modulation agents.

## Figures and Tables

**Table 1.** Summary of redox states and responsive to therapeutic agents of PrEC, PC3, RWPE1, WPE1-NA22 (NA22), WPE1-NB26 (NB26), LNCaP, and LNCaP-C4-2 cells. N/A = Non-applicable.

		**Cell lines**		
**Invasion ability in *in vitro* system cell invasion**		PrEC < PC3	RWPE1 < NA22 < NB26	LNCAP < LNCAP-C4-2
**Intracellular redox state**	**Antioxidant capacity**	PrEC < PC3	RWPE1 < NA22 < NB26	LNCAP < LNCAP-C4-2
	**GSH/GSSG**	PrEC < PC3	RWPE1 < NA22, NB26	LNCAP < LNCAP-C4-2
**Extracellular redox state**	**GSH/GSSG**	PrEC < PC3	RWPE1 < NA22, NB26	LNCAP < LNCAP-C4-2
**Treatment responsiveness**	**TNF**	PrEC < PC3	RWPE1 = NA22 = NB26	N/A
	**TRAIL**	PrEC = PC3	RWPE1 <NA22, NB26	N/A
	**PEITC**	N/A	RWPE1 < NA22 < NB26	N/A
	**R1881**	N/A	N/A	LNCAP < LNCAP-C4-2
